# Fate of the UPR marker protein Kar2/Bip and autophagic processes in fed-batch cultures of secretory insulin precursor producing *Pichia pastoris*

**DOI:** 10.1186/s12934-018-0970-3

**Published:** 2018-08-09

**Authors:** Gustavo Roth, Ana Letícia Vanz, Heinrich Lünsdorf, Manfred Nimtz, Ursula Rinas

**Affiliations:** 1 Technical Chemistry–Life Science, Leibniz University of Hannover, Hannover, Germany; 2 Helmholtz Centre for Infection Research, Inhoffenstrasse 7, 38124 Brunswick, Germany

**Keywords:** Autophagy, Kar2/Bip, *Pichia pastoris*, Unfolded protein response

## Abstract

**Background:**

Secretory recombinant protein production with *Pichia* (*syn. Komagataella*) *pastoris* is commonly associated with the induction of an unfolded protein response (UPR) usually apparent through increased intracellular levels of endoplasmic reticulum (ER) resident chaperones such as Kar2/Bip. During methanol-induced secretory production of an insulin precursor (IP) under industrially relevant fed-batch conditions the initially high level of intracellular Kar2/Bip after batch growth on glycerol unexpectedly declined in the following methanol fed-batch phase misleadingly suggesting that IP production had a low impact on UPR activation.

**Results:**

Analysis of the protein production independent level of Kar2/Bip revealed that high Kar2/Bip levels were reached in the exponential growth phase of glycerol batch cultures followed by a strong decline of Kar2/Bip during entry into stationary phase. Ultra-structural cell morphology studies revealed autophagic processes (e.g. ER phagy) at the end of the glycerol batch phase most likely responsible for the degradation of ER resident chaperones such as Kar2/Bip. The pre-induction level of Kar2/Bip did not affect the IP secretion efficiency in the subsequent methanol-induced IP production phase. During growth on methanol intracellular Kar2/Bip levels declined in IP producing and non-producing host cells. However, extracellular accumulation of Kar2/Bip was observed in IP-producing cultures but not in non-producing controls. Most importantly, the majority of the extracellular Kar2/Bip accumulated in the culture supernatant of IP producing cells as truncated protein (approx. 35 kDa).

**Conclusions:**

Rapid growth leads to higher basal levels of the major UPR marker protein Kar2/Bip independent of recombinant protein production. Entry into stationary phase or slower growth on poorer substrate, e.g. methanol, leads to a lower basal Kar2/Bip level. Methanol-induced secretory IP production elicits a strong UPR activation which counteracts the reduced UPR during slow growth on methanol. The major ER chaperone Kar2/Bip is found together with recombinant IP in the culture medium where full-length Kar2/Bip accumulates in addition to large amounts of truncated Kar2/Bip. Thus, for judging UPR activating properties of the produced protein it is important to additionally analyze the medium not only for intact Kar2/Bip but also for truncated versions of this UPR reporter protein.

**Electronic supplementary material:**

The online version of this article (10.1186/s12934-018-0970-3) contains supplementary material, which is available to authorized users.

## Background

The methylotrophic yeast *Pichia* (*syn. Komagatella*) *pastoris* is a well-established eukaryotic host for the production of heterologous proteins preferentially secreted into the medium to simplify further down-stream procedures [1–3].

Heterologous secretory protein production in *P. pastoris* is often connected to the induction of an unfolded protein response (UPR) leading to increased expression of UPR responsive genes [4–11] and correspondingly elevated intracellular levels of the encoded UPR responsive proteins encompassing endoplasmic reticulum (ER) resident chaperones and foldases [9, 12–14]. The most prominent ER chaperone, Kar2/Bip acts as the sensor protein for the presence of misfolded proteins but is also involved in chaperoning protein folding or targeting recalcitrant proteins to the endoplasmic reticulum associated degradation (ERAD) pathway thereby reducing protein misfolding associated stress in the ER [15]. Co-overproduction of Kar2/Bip in *P. pastoris* has been investigated for enhanced secretion of target proteins with mixed success [11, 16, 17].

The impact of secretory recombinant protein production on UPR activation in *P. pastoris* through proteome analysis can be assessed by analysing the intracellular content of UPR responsive proteins such as Kar2/Bip [9, 12–14, 18]. In most cases, Kar2/Bip increases after induction of recombinant protein production as has been shown for the methanol-induced production of the intracellular ER-retained Hepatitis B surface antigen [13] but also during secretory production of trypsinogen [12] and an anti-CD3 immunotoxin [14]. Interestingly, Kar2/Bip was not identified among many other UPR responsive proteins in *P. pastoris* during secretory xylanase production using the sensitive iTRAQ LC–MS/MS analysis [9].

Surprisingly, a decrease of Kar2/Bip and other UPR related proteins was detected during methanol-induced secretory insulin precursor (IP) production in *P. pastoris* in shake flask but also in industrially relevant fed-batch cultures [18]. Compared to the non-producing host strain the decrease of the intracellular level of Kar2/Bip was less pronounced suggesting that production of IP elicits a detectable but not very strong UPR response which is repressed by a general culture condition dependent UPR down-regulation after the shift from batch growth on glycerol to fed-batch growth on methanol. It was hypothesized that unexpected high levels of intracellular Kar2/Bip at the end of the glycerol batch phase may result from high initial glycerol concentrations, and a correspondingly high medium osmolarity, leading to an efficient secretion of IP during the following methanol-induced IP production phase.

The aim of this study was to analyze more deeply the impact of culture conditions on the basal level of Kar2/Bip and the impact of the preinduction Kar2/Bip level on secretory IP production efficiency. The IP production related UPR analysis also encompassed the analysis of the culture medium for full-length and truncated versions of the UPR marker protein Kar2/Bip. Finally, the UPR analysis was complemented by electron microscopic cell studies to search for autophagic processes.

## Results

### Influence of initial glycerol concentration and growth phase/rate on the protein production independent basal level of Kar2/Bip in batch culture

The extent of the constitutive or basal level of the UPR in the absence of recombinant protein production was determined through analysis of the intracellular level of Kar2/Bip in cells growing on defined medium in controlled bioreactor batch cultures with glycerol as sole carbon source. The initial concentration of glycerol was varied from 30 to 125 g/L and the level of intracellular Kar2/Bip followed during the time course of batch growth.

**Fig. 1:**
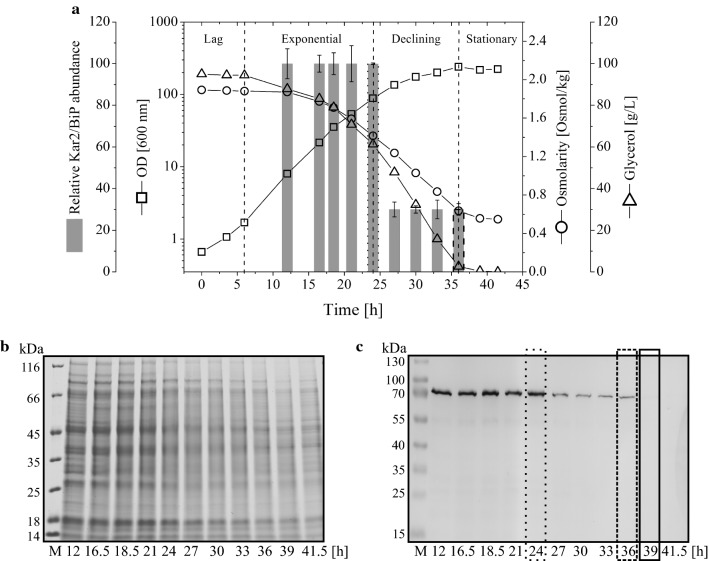
Intracellular Kar2/Bip level during glycerol batch growth of *P. pastoris* X-33 carrying the *aox1* promoter-controlled IP gene. Cells were grown in the 2 L bioreactor in a batch procedure with 95 g/L glycerol as carbon source. **a** The time-dependent relative intracellular Kar2/Bip abundance (gray bars), biomass (optical density: squares), glycerol concentration (triangles), and osmolarity (circles) of the culture broth are given. The dashed vertical lines point to the borders of indicated growth phases. **b** SDS-PAGE gels and **c** corresponding immunoblots probing for Kar2/Bip in total cell lysates. M denotes the molecular weight marker and the numbers on bottom of the lanes the post-inoculation sampling time points. Boxed lanes correspond to boxed bars in (**a**)

Surprisingly, these analyses revealed that intracellular Kar2/Bip levels were independent of the initial glycerol concentrations but highly dependent on the growth phase. Kar2/Bip level were highest during the exponential growth phase but declined rapidly to almost undetectable levels after cells entered into stationary phase suggesting active degradation of Kar2/Bip (Fig. 1). Similar profiles and intracellular Kar2 levels during batch growth were observed at initial glycerol concentrations ranging from 30 to 125 g/L (Table 1, Additional file 1).

**Table 1:** Growth performance and intracellular Kar2/Bip level of *P. pastoris* X-33 carrying the *aox1* promoter-controlled IP gene during batch growth in a 2 L bioreactor with different initial glycerol concentrations

Glycerol (g/L)	30	60	95	125
µ_max_ (1/h)	0.26	0.19	0.18	0.18
Osmolarity (Osmol/kg)	0.8	1.4	1.9	2.5
Relative Kar2/Bip abundance	114	123	100	116

### Autophagic processes in *P. pastoris* entering stationary phase during growth on glycerol

**Fig. 2:**
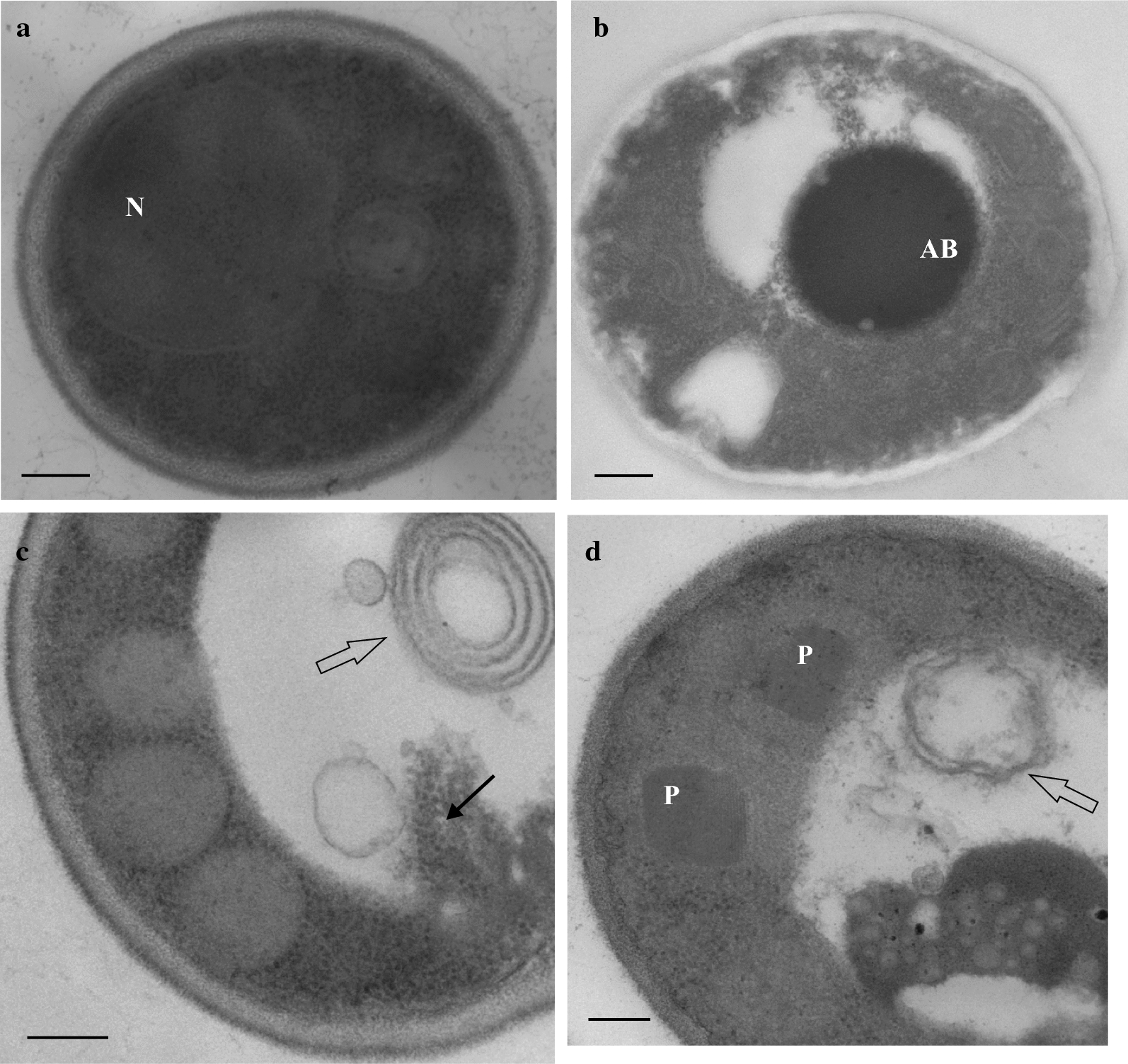
Autophagic processes in *P. pastoris* approaching stationary phase in glycerol batch cultures and during methanol-induced IP production in fed-batch cultures. **a**–**c** Cells were first grown in a 15 L bioreactor in batch mode with 95 g/L glycerol as carbon source as described previously [19]. Directly after depletion of glycerol and prior to methanol feeding cells were examined by transmission electron microscopy. Representative images of cells from the morphologically diverse population are shown. **d** After depletion of glycerol IP production was induced by methanol feeding as described before [19]. Representative image taken 48 h after the onset of methanol feeding. N: nucleus, AB: autophagic body, P: peroxisome, open arrows: membranous structures with the typical features of the delimiting ER membranes (ER-phagy), black arrow: ribosomes (ribophagy). For typical features of ER-phagy in electron micrographs of *S. cerevisiae* [32–34] and *P. pastoris* [29] please consult figures in indicated references. Bars represent 200 nm

The sudden and strong decline of Kar2/Bip at the end of the glycerol batch phase indicated active degradation presumably caused by autophagic processes during entry into stationary phase. Autophagic processes are known to occur in glycerol grown *P. pastoris* during entry into stationary phase where around 20% of cells were observed to have vacuoles with embedded autophagic bodies [13]. An electron microscopic survey of IP producing cells at the end of the glycerol batch phase prior to methanol-induced IP production also revealed autophagic cells amongst others also cells at the beginning of ER- and ribophagy (Fig. 2). Autophagic processes involving ER- and ribophagy at the end of the glycerol batch are not unique to the IP producing strain but were also observed at the end of the glycerol batch phase of a GS115 derived HBsAg production strain (data not shown, [13]).

### Effect of pre-induction Kar2/Bip level on methanol induced secretory IP production kinetics and final IP concentrations

To test if the growth phase, or more specifically, the Kar2/Bip level at the initiation of methanol-induced IP production, correlates positively with the efficiency of IP secretion, induction of IP synthesis was carried out through transfer of cells from different growth phases of the glycerol bioreactor batch culture into shake flask cultures containing complex methanol induction medium. IP production was carried out under conditions of pulsed methanol feeding.

**Fig. 3:**
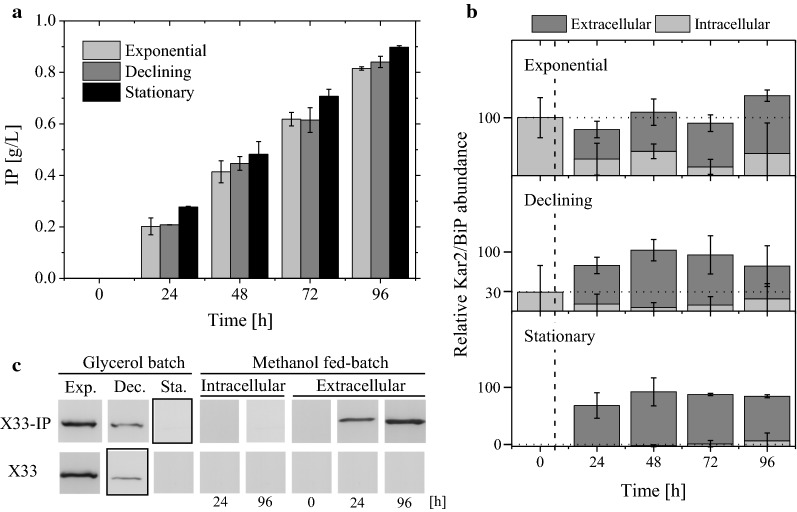
Effect of pre-induction Kar2/Bip content on methanol induced IP production and post-induction Kar2/Bip level. Cells were first grown in the 10 L bioreactor in a batch procedure with 95 g/L glycerol as carbon source. Subsequently, they were subjected to methanol-induced IP production in shake flask fed-batch cultures starting either with cells from the exponential, declining, or stationary growth phase of the batch culture. **a** Methanol-induced secretory IP production and **b** relative intra- and extracellular pre- and post-induction Kar2/Bip abundance. **c** Comparative immunoblots probing for intra- and extracellular Kar2/Bip from samples of IP producing and host cells taken from bioreactor glycerol batch and shake flask methanol fed-batch cultures. The bands inside the highlighted rectangles indicate the intracellular Kar2/Bip level prior to methanol addition

These experiments clearly revealed that the kinetics of IP secretion as well as the final concentrations of extracellular IP were independent of the pre-induction growth phase, or more specifically, they were independent on the Kar2/Bip level at the onset of methanol induced IP production (Fig. 3a). These experiments also clearly proved that a high Kar2/Bip level at the onset of methanol-induced IP production did not correlate with more efficient IP secretion (Fig. 3a). On the contrary, final IP concentrations were even slightly higher (Fig. 3a) when induction was carried out using late stationary phase cells which contained only negligible amounts of Kar2/Bip as determined by Western blot analysis and subsequent immunostaining (Fig. 3b, c).

### Secretory IP production results in extracellular accumulation of the UPR marker protein Kar2/Bip

For studies on the post-induction UPR, the time-course of intracellular Kar2/Bip levels were determined after the start of the methanol-induced IP production phase. In all experimental setups, intracellular Kar2/Bip reached or kept a low, almost undetectable level after transfer to methanol containing induction medium (Fig. 3b, c). Interestingly, in all IP producing cultures also extracellular accumulation of Kar2/Bip was observed (Fig. 3b, c). Control experiments with the host strain using identical culture conditions did not lead to the extracellular accumulation of Kar2/Bip clearly proving that Kar2/Bip secretion was connected to secretory IP production (Fig. 3c). Moreover, no extracellular Kar2/Bip was detected during the glycerol batch phase neither in the host nor in the production strain corroborating the correlation of extracellular Kar2/Bip accumulation to IP secretion (Fig. 3c). A quantitative analysis of Kar2/Bip additionally revealed that the extracellular Kar2/Bip resulted from de novo synthesis during the methanol phase and not from Kar2/Bip already formed before in the glycerol batch phase (Fig. 3b).

### The fate of Kar2/Bip in controlled bioreactor fed-batch cultures

**Fig. 4:**
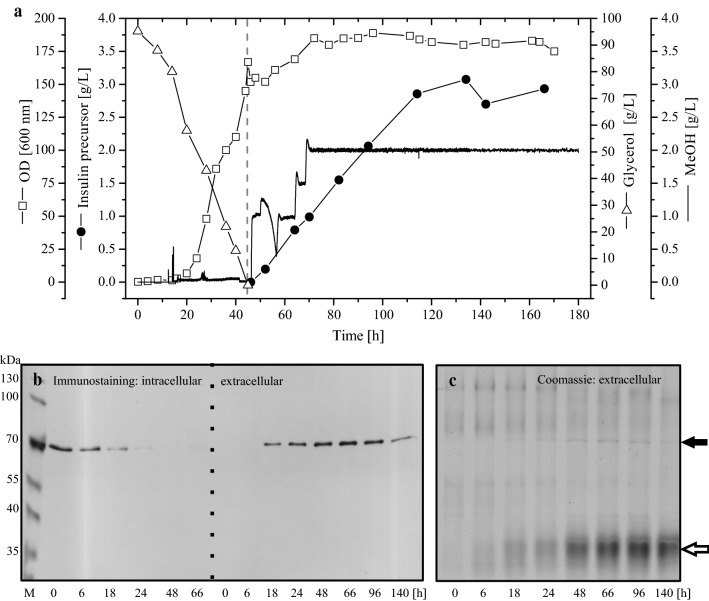
Post-induction level of intra- and extracellular Kar2/Bip in controlled bioreactor fed-batch culture. Cell growth and IP production were carried out in controlled bioreactor fed-batch culture as described previously [19]. **a** Concentrations of glycerol (open triangles), biomass (optical density: open squares), IP (filled circles), and methanol (solid line) are given. The dashed vertical line indicates the end of the glycerol batch (pre-induction) and the start of the methanol feeding phase (post-induction). **b** Immunoblot probing for the time-dependent change of intra- and extracellular Kar2/Bip in post-induction samples. **c** Extracellular proteins detected by Coomassie blue staining of SDS-PAGE gel indicating proteins identified by mass spectrometry. The positions of full-length Kar2/Bip (~ 74 kDa, filled arrow) and the truncated approximately centrally cleaved Kar2/Bip fragment (~ 35 kDa, open arrow) are indicated. **b**, **c** M denotes the molecular weight marker and the numbers on the bottom of the lanes the post-induction sampling time points. Please note the IP (7 kDa) is not detectable on conventional SDS-PAGE gels (see also [19]). Please also note that immunoblot and SDS-PAGE gel are aligned for the same molecular weight scale

Detection of Kar2/Bip in the medium of IP-producing cells in shake flask cultures under conditions of pulsed methanol feeding prompted us to re-analyze UPR induction during IP production in industrially relevant controlled fed-batch cultures by focussing on the analysis for Kar2/Bip in cell free medium samples. Cells were initially grown on 95 g/L glycerol, after depletion of glycerol secretory IP production was initiated by methanol addition to a final concentration of 2 g/L which was kept constant by continuous methanol feeding (Fig. 4a, [19]).

Analysis of the intracellular Kar2/Bip level during the methanol-induced IP production phase revealed the expected time-dependent decrease of Kar2/Bip as reported previously (Fig. 4b, [18]). In addition, extracellular accumulation of Kar2/Bip was observed in the culture broth detectable by Western blot analysis with subsequent immunostaining (Fig. 4b). Moreover, Kar2/Bip was also detectable as a faint band in Coomassie stained gels (Fig. 4c). The identity of the faint band at approx. 70 kDa in Coomassie stained gels as Kar2/Bip was verified by Maldi-ToF analysis (data not shown). Interestingly, Coomassie stained gels of cell free culture samples from IP producing cells of controlled fed-batch cultures also revealed a strong single broad band corresponding to protein(s) of a molecular mass of approx. 35 kDa which were not reacting with antibodies against the ER retention signal HDEL (Fig. 4a, see also Fig. 3 in [19]). As the culture medium of *P. pastoris* usually does not contain endogenous *Pichia* proteins efforts were undertaken to identify these protein(s). Attempts by Maldi-ToF analysis were repeatedly unsuccessful.

Finally, the protein band of approx. 35 kDa was subjected to HPLC–MS/MS analysis after in-gel tryptic digestion and mass spectrometric sequencing of the resulting peptides. These analyses revealed the presence of truncated Kar2/Bip as dominant component of this protein band (for details see also Additional file 2). Full-length mature Kar2/Bip has a molecular mass of approx. 74 kDa, thus the truncated Kar2/Bip is only half of its original size. Since all Kar2/Bip peptides from the N-terminal part of the protein were of higher intensity than peptides originating from the C-terminal part of the protein, it can be concluded that the C-terminal part of Kar2/Bip is for the most part degraded by proteases. This is in agreement with the failure to detect Kar2/Bip by immunostaining in the extracellular protein fraction, since the antibody used detects the ER retention signal which is located at the carboxyterminus. In addition to peptides originating from Kar2/Bip, other peptides of (truncated) *Pichia* proteins were detected within the tryptic digest of this protein band including the ER-derived protein disulphide isomerase Pdi (58 kDa full size) and also the vacuolar proteinase B (YscB, 52 kDa full size) (for a detailed list of all identified proteins see also Additional file 2.

## Discussion

Only few studies address the impact of environmental conditions on the extent of basal, misfolded protein independent UPR in yeast. For *Saccharomyces cerevisiae* it has been shown that—apart from aberrant protein secretion—the extent of the UPR is also connected to the nutritional state of the cell [20–22]. For example, addition of ammonium to nitrogen-starved *S. cerevisiae* causes UPR activation [22]. Other environmental conditions such as medium osmolarity and temperature also affect UPR as has been shown for non-producing and heterologous protein secreting *P. pastoris* in chemostat cultures [23, 24].

Our findings show that rapid growth during the exponential phase in batch culture is connected to an activated or higher basal level of the UPR marker protein Kar2/Bip (Fig. 1). Entry into stationary phase is linked to growth rate decrease which appears to be responsible for UPR downregulation as indicated by a declining Kar2/Bip level. UPR activation during rapid growth is independent of recombinant protein production and presumably due to the enhanced ER-associated endogenous yeast protein synthesis as it has been observed in the absence of secretory recombinant protein production and with the host not even carrying a recombinant gene (Fig. 3). The strong decline of the UPR sensor protein Kar2/Bip during entry into stationary phase also suggests active degradation of Kar2/Bip most likely through autophagic processes which are involved in the recycling of damaged or redundant cellular components. Electron microscopy studies gave clear evidence for autophagy in *P. pastoris* at the end of the glycerol batch phase and also during the methanol-induced IP production phase (Fig. 2). The electron micrographs of cells from the end of the glycerol batch phase revealed a yeast population with a diverse morphology including cells without visible vacuoles (Fig. 2a), cells with vacuoles containing autophagic bodies (Fig. 2b) and also cells at the onset of ribo- and ER-phagy with vacuoles starting to upload still intact ribosomes and double membranous structures with the typical features of the delimiting ER membranes (Fig. 2c). Thus, evidence for the strong decline of the ER resident marker protein Kar2/Bip at the end of the glycerol batch phase and concurrent detection of ER-phagy strongly suggest that the decreasing level of Kar2/Bip can be attributed to nutritional downshift induced autophagy.

Interestingly, we did not observe a higher basal level of the UPR marker protein Kar2/Bip with a higher initial glycerol concentration during batch growth. Instead, the intracellular level of Kar2/Bip remained unaffected by the initial glycerol concentration (Table 1, Additional file 1). Higher glycerol concentrations cause an increase in the medium osmolarity (Table 1) and it was reported previously that an increase in the medium osmolarity through salt addition resulted in enhanced levels of the UPR proteins Kar2/Bip and Pdi in non-producing *P. pastoris* during carbon limited growth in continuous cultures [24]. The independence of the Kar2/Bip level on increasing initial glycerol concentrations in batch culture might be explainable through the concurrent decrease of the maximum growth rate (Table 1) counteracting UPR activation through elevated medium osmolarity. Down-regulation of UPR responsive genes at lower growth rate in glucose-limited chemostat cultures of *P. pastoris* producing a *GAP* promoter controlled recombinant protein has been observed before [25]. Thus, slower growth at higher initial glycerol concentrations might reduce UPR activation through elevated medium osmolarity. Also, the basal recombinant protein production independent UPR—as judged by the intracellular level of Kar2/Bip—was higher during exponential growth on glycerol than during slow growth on methanol (see host strain X-33 in Fig. 3c) confirming that slower growth is connected to a lower basal level of the UPR marker protein Kar2/Bip.

On the other hand, methanol-induced secretory IP production counteracts the UPR down-regulation during slow growth on methanol and results in de novo Kar2/Bip synthesis followed by an IP secretion dependent release of Kar2/Bip into the culture medium. Accumulation of full-length Kar2/Bip in the culture medium was detectable by Western blot analysis (Figs. 3 and 4) and even by conventional SDS-PAGE analysis and Coomassie blue staining (Fig. 4) suggesting a substantial amount of extracellular accumulation. Release of Kar2/Bip in cultures of *P. pastoris* has been detected before during the secretory production of two other recombinant proteins using Western blot analysis [14, 17]. In these experiments it was also shown that traces of Kar2/Bip were found in the medium in the absence of secretory protein production when growth occurred in rich medium [14]. In addition, overexpression of spliced *HacI*, the transcription factor controlling the expression of UPR genes, was also leading to extracellular accumulation of Kar2/Bip in *P. pastoris* [26]. It has been suggested for the yeast *Saccharomyces cerevisiae*, that, regardless of the initial cause, strong UPR activation appears to be the critical factor for extracellular release of Kar2/Bip [27]. In our experiments we did not detect Kar2/Bip in the medium in the absence of secretory IP production (Fig. 3, data not shown). However, during methanol-induced IP production the majority of Kar2/Bip was found in the medium (Figs. 3 and 4) suggesting a strong UPR-inducing impact of secretory IP production.

The detection of Kar2/Bip in the culture medium may indicate that Kar2/Bip does not only promote folding in the ER or transfer of aberrant proteins to the ERAD pathway but may also accompany some (aberrant) proteins on the entire passage through the secretory pathway to the cell exterior. Binding of Kar2/Bip to another recombinant protein has been verified by pull down assay [28], thus, a potential interaction of IP and Kar2/Bip might be responsible for the concurrent appearance of both proteins in the culture medium. On the other hand detection of large amounts of truncated versions of the ER-resident proteins Kar2/Bip and Pdi as well as the vacuolar protease B in the culture medium may also indicate that IP production mediated UPR stress enhances ER-specific autophagy possibly leading to the extracellular release of vacuolar content. Upregulation of genes involved in ER-phagy [7] and ER autophagosomes [29] have been detected under conditions of ER stress resulting from the secretory production of recombinant proteins. Also, electron micrographs of IP producing cells from the methanol fed-batch phase of controlled bioreactor cultures did not only reveal evidence for pexophagy but also indications for ER-phagy (Fig. 2d). Thus, accumulation of ER resident chaperones such as Kar2/Bip in the culture medium under conditions of ER-stress may not only result from (co)secretion but could also arise through the release of vacuolar content into the culture medium.

## Conclusions

Rapid growth leads to a higher basal level of the UPR marker protein Kar2/Bip independent of recombinant protein production. Entry into stationary phase or slower growth on poorer substrate, e.g. methanol, leads to a lower level of Kar2/Bip. Methanol-induced secretory IP production elicits a strong UPR activation which counteracts the reduced UPR during slow growth on methanol. The major ER chaperone Kar2/Bip is found together with recombinant IP in the culture medium where full-length Kar2/Bip accumulates in addition to large amounts of truncated Kar2/Bip. Thus, for judging UPR activating properties of the produced protein it is important to additionally analyze the medium not only for intact Kar2/Bip but also for truncated versions of this UPR reporter protein.

## Methods

### Strains and growth conditions

#### Strains

The *P. pastoris* host strain X-33 was from Invitrogen (Carlsbad, CA). Details of the construction of the recombinant *P. pastoris* strain X-33 carrying a codon-optimized copy of a synthetic IP gene for secretory IP production under the control of the *aox1* promoter (Mut^+^) and usage of the α-factor secretory signal are given elsewhere [19].

#### Precultures

A starter culture was prepared in a 500 mL sterile baffled shake flask containing 100 mL of yeast nitrogen base with ammonium sulfate and without amino acids (1.34 g/L), glycerol (20 g/L) and biotin (0.4 mg/L) from glycerol stock cultures (initial OD_600_ 0.03, 30 °C, 160 rpm, 18 h to OD_600_ ~ 5). The starter culture was used to inoculate 500 mL of defined medium in a 2 L baffled shake flask (starting OD_600_ 0.1, 30 °C, 160 rpm, 20 h to OD_600_ ~ 10). The defined medium was identical to the medium employed for the glycerol batch phase in bioreactor cultures [19, 30] except that the glycerol concentration was 25 g/L.

#### Bioreactor batch cultivations

Bioreactor cultivations were started with an initial OD_600_ 0.5. Cells were grown either in a 2 L Biostat B plus or in a 10 L Biostat C (B. Braun Biotech International, Germany) using a defined medium as described previously [19] except that glycerol concentrations were varied between 30 and 125 g/L. Details are specified in the figure captions.

#### Shake flask fed-batch cultivations

After bioreactor batch growth in defined medium containing glycerol cells were pelleted by centrifugation (30 min, 4 °C, 3347×*g*), washed with sterile phosphate buffered saline (PBS; 137 mmol/L sodium chloride, 2.7 mmol/L potassium chloride, 10 mmol/L disodium hydrogen phosphate and 1.8 mmol/L potassium phosphate), re-centrifuged and re-suspended in 150 mL baffled shake flasks containing 35 mL buffered methanol complex medium (BMMY) with 1% (w/v) yeast extract, 2% (w/v) peptone, 100 mmol/L potassium phosphate (pH 6.0), 1.34 g/L yeast nitrogen base with ammonium sulfate and without amino acids and 0.4 mg/L biotin to an OD600 ~ 100 (30 °C, 160 rpm, addition of 1% (v/v) methanol twice a day at 12 h intervals for a total period of 96 h). Details are specified in the figure captions.

#### Bioreactor fed-batch cultivations

Growth and IP production under industrially relevant conditions using a defined medium were carried out as described before [19, 30]. Cells were grown in a 15 L Biostat C (B. Braun Biotech International, Germany) using glycerol as sole carbon source with an initial glycerol concentration of 95 g/L. After depletion of glycerol, IP production was induced by a pulsed methanol addition and subsequent methanol feeding to maintain the methanol concentration at 2 g/L.

### SDS-PAGE and immunodetection

#### Sample preparation

Cells were collected by centrifugation (13,000 rpm for 5 min) and re-suspended in lysis buffer (25 mmol/L potassium phosphate buffer pH 8.0, 5 mmol/L EDTA, 8% (w/v) glycerol, 500 mmol/L sodium chloride) to an OD_600_ 50. For cell disruption, aliquots of this suspension were diluted 1:1 with loading buffer (20 mmol/L Tris–HCl pH 8.0, 2 mmol/L EDTA, 5% (w/v) SDS, 0.02% (w/v) bromophenol blue, 5.5% (v/v) glycerol, 20% (v/v) 2-mercaptoethanol) and boiled for 1 h at 96 °C. Medium samples were diluted 1:1 with modified loading buffer (as above but with 10% (v/v) 2-mercaptoethanol) and boiled at 96 °C for 5 min.

#### SDS-PAGE, Western blotting and immunostaining

Samples (loading volume 7 μL) were run on 12% SDS-PAGE gels prior to Coomassie Brilliant Blue staining or electroblotting onto PVDF membranes (Bio-Rad, Hercules, USA) at 15 V for 45 min. The membranes were blocked with 5% (w/v) skimmed milk (Difco, France), 2% (w/v) polyvinylpyrrolidone (Carl Roth, Germany) in PBS containing 1% (v/v) Tween 20 (PBS-T) for 2 h. After washing the membranes with PBS-T, the mouse anti-HDEL antibody (2E7) (sc-53472; 1:1000 dilution, Santa Cruz Biotechnology, USA) was added and the membranes incubated for 1 h at room temperature. After washing with PBS-T, the secondary anti-mouse antibody (1:5000 dilution, Calbiochem, Germany) was added and incubation continued for 1 h. Immunostaining was done using 3,3′,5,5′ tetramethylbenzidine (Sigma, Germany) as substrate. The mouse anti-HDEL antibody (2E7) is a commercial antibody raised against a synthetic HDEL peptide corresponding to the C-terminus of yeast BiP (also designated GRP 78) which should bind only to the six *P. pastoris* proteins containing the C-terminal HDEL sequence, namely Kre5 (166.2 kDa), Sec12 (116.2 kDa), Lhs1 (99.5 kDa), Kar2/Bip (74.2 kDa), Pdi1 (57.8 kDa), and Mpd1 (33.5 kDa) [28].

For relative quantification, the intracellular Kar2/Bip abundance was normalized by densitometry analysis of immunostained membranes and SDS-PAGE gel images using ImageJ (National Institutes of Health, USA). For comparison, all samples were run on duplicate 12% SDS-PAGE gels. One gel was stained with Coomassie Brilliant Blue and the second gel used for Western blotting and immunostaining. The Kar2/Bip Western blot relative abundance was determined as the quotient of the Kar2/Bip band area on the immunostained membrane and the total protein content in the corresponding sample of the Coomassie Brilliant Blue stained SDS-PAGE gel. The intracellular mid-exponential sample of the 95 g/L glycerol batch was used as internal standard on each gel and set to the relative Kar2/Bip abundance 100.

### Mass spectrometry for protein identification (LC–MS/MS)

The peptides extracted after tryptic in-gel digestion of the relevant band from Coomassie stained gels were analyzed on an UltiMate 3000 RSLC nano LC system connected to an Orbitrap Fusion mass spectrometer (Thermo Scientific). MS survey scans were alternated with MS/MS scans of daughter ion spectra obtained from peptide components by higher collision energy induced dissociation (HCD). All spectra were recorded using the orbitrap detector with a resolution of 120 k for survey scans and 15 k for daughter ion spectra. The experimental setup was as follows: the peptide mixture was loaded onto a 75 μm × 2 cm precolumn (Acclaim PepMap 100, C18, 3 μm, Thermo Scientific) and eluted over 60 min using a 75 μm × 50 cm analytical column (Acclaim PepMap RSLC, C18, 2 μm, Thermo Scientific) with a gradient buffer 0–95% acetonitrile in 0.1% formic acid. The data were acquired using the Xcalibur 2.3 software (Thermo Scientific) and processed with the Peaks 6 software (Bioinformatics Solution). MS/MS data were searched versus the *Pichia* (*syn. Komagataella*) *pastoris* database extracted from the NCBI non redundant database.

### Transmission electron microscopy

Culture samples were immediately fixed at ambient temperature in 2.5% (v/v) glutardialdehyde in 20 mmol/L HEPES buffer (pH 7.1) for 30 min. Further processing and image analysis was carried out as described previously [13, 31].

### Other analytical procedures

Cell growth was followed by measurement of the optical density at 600 nm (OD_600_) using a Multiskan GO UV–Vis spectrophotometer (Thermo Fisher Scientific, Germany). The glycerol concentration was analyzed by high performance liquid chromatography (Chromaster system, Hitachi, USA) with an Aminex HPX-87H column (Bio-Rad Laboratories, USA) at 60 °C using 5 mmol/L H_2_SO_4_ as the mobile phase at a flow rate of 0.6 mL/min. The osmolarity of the medium was determined using an Osmomat 3000 (Gonotec, Germany) freezing-point osmometer. Quantification of IP was carried out essentially as described previously [19] with minor modifications. Filtered aliquots of cell-free supernatants were mixed 1:1 with solution A (0.15% (v/v) trifluoroacetic acid in ultrapure water) and analyzed by reversed-phase high performance liquid chromatography (RP-HPLC) using a 3 μm SUPELCOSIL™ LC-304 (Sigma-Aldrich, USA) column (3.3 cm × 4.6 mm) at 24 °C with a Chromaster liquid chromatography system (Hitachi, USA). Elution was performed with a gradient formed by mixing solutions A and B (0.15% (v/v) trifluoroacetic acid in acetonitrile) as follows: 10% B (0–6 min), 10–43% B (6–41 min), 43–100% B (41–43 min), 100–10% B (43–53 min), 10% B (53–60 min). The flow rate was maintained at 1 mL/min and the column effluent was monitored at 214 and 280 nm. Freeze-dried purified IP was used as standard (concentration determined by weight and by Pierce bicinchoninic acid kit, Thermo Fisher Scientific, Germany).

## Additional files

**Additional file 1.** Growth performance and intracellular Kar2/Bip level of *P. pastoris* X-33 carrying the *aox1* promoter-controlled IP gene during batch growth in a 2 L bioreactor with different initial glycerol concentrations (30 to 125 g/L). (A) The time-dependent changes in the biomass (top), glycerol concentration (middle), and osmolarity of the culture broth (bottom) are given (initial glycerol concentration 30 g/L, open squares; 60 g/L, full squares; 95 g/L, open circles; and 125 g/L full circles). B) SDS-PAGE gels and (C) corresponding immunoblots probing for Kar2/Bip in total cell lysates (all samples from the mid-exponential growth phase). The numbers on top of the lanes denote the initial glycerol concentration and “r” the repetition batch experiment. M denotes the molecular weight marker and the other numbers on the bottom of the lanes the batch culture post-inoculation sampling time points.

**Additional file 2.** HPLC–MS/MS analysis of protein band at approx. 35 kDa.

## Abbreviations

ER: endoplasmic reticulum
ERAD: endoplasmic reticulum associated degradation
HBsAg: hepatitis B surface antigen
IP: insulin precursor
iTRAQ LC–MS/MS: isobaric tags for relative and absolute quantitation liquid chromatography–tandem mass spectrometry
Maldi-ToF: matrix-assisted laser desorption/ionization-time-of-flight
NCBI: National Center for Biotechnology Information
PBS: phosphate buffered saline
SDS-PAGE: sodium dodecyl sulfate polyacrylamide gel electrophoresis
UPR: unfolded protein response
