# Supplementary material for: Fate of the UPR marker protein Kar2/Bip and autophagic processes in fed-batch cultures of secretory insulin precursor producing Pichia pastoris
Source: Microb Cell Fact. 2018 Aug 9;17:123. doi: 10.1186/s12934-018-0970-3 (PMC6083527; doi:10.1186/s12934-018-0970-3)
Supplement: Supplementary file 1 — Additional file 1. Growth performance and intracellular Kar2/Bip level of P. pastoris X-33 carrying the aox1 promoter-controlled IP gene during batch growth in a 2 L bioreactor with different initial glycerol concentrations (30 to 125 g/L). (A) The time-dependent changes in the biomass (top), glycerol concentration (middle), and osmolarity of the culture broth (bottom) are given (initial glycerol concentration 30 g/L, open squares; 60 g/L, full squares; 95 g/L, open circles; and 125 g/L full circles). B) SDS-PAGE gels and (C) corresponding immunoblots probing for Kar2/Bip in total cell lysates (all samples from the mid-exponential growth phase). The numbers on top of the lanes denote the initial glycerol concentration and “r” the repetition batch experiment. M denotes the molecular weight marker and the other numbers on the bottom of the lanes the batch culture post-inoculation sampling time points. [file 12934_2018_970_MOESM1_ESM.doc]

**Fate of the UPR marker protein Kar2/Bip and autophagic processes in fed-batch cultures of secretory insulin precursor producing *Pichia pastoris***

Gustavo Roth^1^, Ana Letícia Vanz^1^, Heinrich Lünsdorf^2^, Manfred Nimtz^2^, and Ursula Rinas^1,2*^


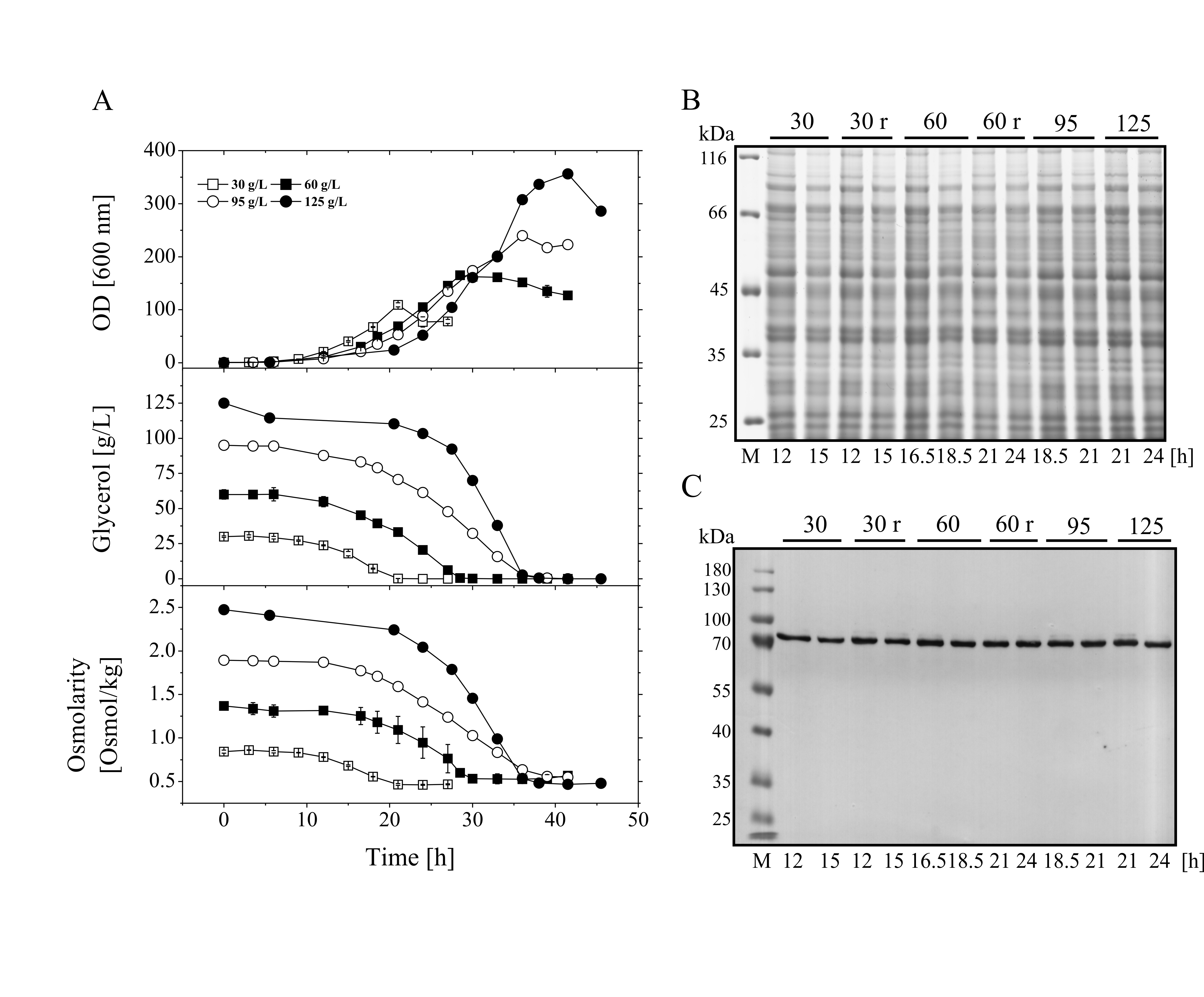


**Additional file 1: Fig S1.** Growth performance and intracellular Kar2/Bip level of *P. pastoris* X-33 carrying the *aox1* promoter-controlled IP gene during batch growth in a 2 L bioreactor with different initial glycerol concentrations (30 to 125 g/L). (A) The time-dependent changes in the biomass (top), glycerol concentration (middle), and osmolarity of the culture broth (bottom) are given (initial glycerol concentration 30 g/L, open squares; 60 g/L, full squares; 95 g/L, open circles; and 125 g/L full circles). B) SDS-PAGE gels and (C) corresponding immunoblots probing for Kar2/Bip in total cell lysates (all samples from the mid-exponential growth phase). The numbers on top of the lanes denote the initial glycerol concentration and “r” the repetition batch experiment. M denotes the molecular weight marker and the other numbers on the bottom of the lanes the batch culture post-inoculation sampling time points.
